# Dosimetric impact of positional uncertainties and a robust optimization approach for rectal intensity‐modulated brachytherapy

**DOI:** 10.1002/mp.17800

**Published:** 2025-03-31

**Authors:** Björn Morén, Alana Thibodeau‐Antonacci, Jonathan Kalinowski, Shirin A. Enger

**Affiliations:** ^1^ Medical Physics Unit Department of Oncology McGill University Montreal Québec Canada; ^2^ Department of Mathematics Linköping University Linköping Sweden; ^3^ Lady Davis Institute for Medical Research Jewish General Hospital Montreal Québec Canada

**Keywords:** intensity modulated brachytherapy, rectal cancer, robust optimization

## Abstract

**Background:**

Intensity‐modulated brachytherapy (IMBT) employs rotating high‐Z shields during treatment to decrease radiation in certain directions and conform the dose distribution to the target volume. Prototypes for dynamic IMBT have been proposed for prostate, cervical, and rectal cancer.

**Purpose:**

We considered two shielded applicators for IMBT rectal cancer treatment and investigated how rotational uncertainties in the shield angle and translational uncertainties in the source position affect plan evaluation criteria.

**Methods:**

The effect of rotational errors of $3^{\circ }$, $5^{\circ }$ and $10^{\circ }$, and translational errors of 1, 2 and 3 mm on evaluation criteria were investigated for shields with $180^{\circ }$ and $90^{\circ }$ emission windows. Further, a robust optimization approach based on quadratic penalties that includes scenarios with errors was proposed. The extent to which dosimetric effects of positional errors can be mitigated with this model was evaluated compared to a quadratic penalty model without scenarios with errors. A retrospective rectal cancer data set of ten patients was included in this study. Treatment planning was performed using the Monte Carlo‐based treatment planning system, RapidBrachyMCTPS.

**Results:**

For the largest investigated rotational error of $\pm 10^{\circ }$, the clinical target volume $D_{90}$ remained, on average, within 5% of the result without error, while the contralateral healthy rectal wall experienced an increase in the mean $D_{0.1cc}$, $D_{2cc}$, and $D_{50}$ of 26%, 9%, and 1% for the $180^{\circ }$ shield and of 32%, 9%, and 2% for the $90^{\circ }$ shield. For translational errors of ±2 mm, there were increases in dosimetric indices for both the superior (sup) and inferior (inf) dose spill regions. Specifically, for the $180^{\circ }$ shield, the $D_{0.1cc}$, $D_{2cc}$, and $D_{50}$ increased by 13%, 11%, and 10%, respectively, for the sup region, and by 26%, 15%, and 11%, respectively, for the inf region. Similar results were obtained with the $90^{\circ }$ shield. Overall, the robust and traditional models had similar results. However, the number of active dwell positions obtained with the robust model was larger, and the longest dwell time was shorter.

**Conclusions:**

We have quantified the effect of rotational shield and translational source errors of various magnitudes on evaluation criteria for rectal IMBT. The robust optimization approach was generally not able to mitigate positional errors. However, it resulted in more homogeneous dwell times, which can be beneficial in conventional high‐dose‐rate brachytherapy to avoid hot spots around specific dwell positions.

## 1 INTRODUCTION

Worldwide, colorectal cancer is the third most common cancer, with more than 1.9 million new cases in 2020. Approximately one‐third of all cases originate in the rectum.^1^ Total mesorectal excision is the gold standard for curative resection of advanced local tumors, resulting in decreased local recurrence rates and increased overall survival.^2^ Neoadjuvant chemoradiotherapy is recommended for most patients with stage II or III rectal cancer. Preoperative irradiation may reduce the tumor volume, facilitating resection and increasing the possibility of sphincter preservation.^3^ External beam radiotherapy is the most used form of neoadjuvant radiotherapy but is associated with higher rates of postoperative complications.^4^ To reduce radiation‐related toxicities, high‐dose‐rate brachytherapy (HDR BT) is an alternative downstaging modality that results in comparable local control.^5^, ^6^ It can be administered alone or as a boost to external beam radiotherapy for elderly patients with small tumors or patients unfit to undergo surgery.^7^

Intensity‐modulated brachytherapy (IMBT) is a form of HDR BT in which static or dynamic high‐density shields are placed inside the applicator or source to modulate the dose distribution.^8^ In our clinic, endorectal static‐shield IMBT is performed with the flexible intracavitary mold applicator provided by Elekta (Elekta Brachytherapy, Veenendaal, The Netherlands).^9^, ^10^ This cylindrical applicator has an 8mm central cavity that can hold a lead or tungsten alloy rod of the same dimensions to decrease the dose to the contralateral healthy tissues when treating noncircumferential tumors. Webster et al. studied the potential benefits of dynamic modulated brachytherapy for treating rectal cancer.^11^ Their system uses a cylindrical tungsten alloy shield measuring 19mm in diameter and 45mm in length with a $45^{\circ }$ emission window. During treatment, a robotic arm controls the shield rotation. When compared to the intracavitary mold applicator with static shielding, the dose heterogeneity index decreased by 40%, and the $D_{98\%}$ decreased by 40% to 60% for all critical structures.

Treatment optimization usually involves a treatment planning system that considers dose constraints to the tumor and surrounding organs at risk (OAR). The dose‐volume histogram (DVH) and associated dosimetric indices represent the most widely used criteria for evaluating treatment plans. A common example of a dosimetric index is the $D_{X}$ (or $D_{Xcc}$), which corresponds to the minimum dose received by the hottest X% (or X cc) of the structure. Modeling of such criteria yields a mixed‐integer program that has been proposed for treatment planning of HDR BT.^12^, ^13^, ^14^, ^15^, ^16^ However, because of the large number of binary variables, mixed‐integer program techniques generally have long run times, which limits their implementation in clinical practice. Antaki et al. developed a fast mixed integer optimization (FMIO) algorithm to reduce the problem size without loss of precision.^16^ This optimization technique was implemented in RapidBrachyMCTPS, a Monte Carlo‐based treatment planning system.^17^, ^18^ Other methods for treatment planning include inverse planning simulated annealing ^19^ and hybrid inverse planning and optimization.^20^ Both algorithms have been incorporated into commercial treatment planning systems due to their short run times, but the clinically used evaluation criteria based on DVHs are not explicitly included in their models. See Morén et al.^21^ for an overview of optimization models for treatment planning of HDR BT.

Robust optimization approaches account for uncertainties during radiotherapy planning. They have been implemented for intensity‐modulated radiotherapy as well as intensity‐modulated proton therapy.^22^, ^23^ For prostate HDR BT, a method that incorporates target and OAR delineation uncertainties based on multiple delineation scenarios has been proposed.^24^ Other studies have considered uncertainties related to organ volume reconstruction,^25^ as well as uncertainties involving both catheter placement and organ delineation.^26^ Again, for prostate, some authors have investigated patient‐specific catheter shift error thresholds with robust optimization.^27^ Additionally, robust optimization has been investigated for cervical HDR BT.^28^

In the case of dynamic IMBT, it is particularly important to account for uncertainties related to the use of new rotating shields during treatment planning. Inaccuracies in shield rotation could result in parts of the tumor being blocked, thereby affecting the delivered dose, or in parts of the radiation‐sensitive surrounding tissue being irradiated, causing side effects. As organ preservation approaches such as nonoperative treatments and local excision gain momentum in the management of rectal cancer,^29^, ^30^ monitoring and limiting the dose to healthy rectal mucosa becomes increasingly critical. We have previously determined the dosimetric impact of rotational errors for prostate IMBT and developed a robust optimization model.^31^ In continuation of the prior study, this work aimed to evaluate the dosimetric effects of rotational uncertainties in the shield angle and translational uncertainties in the source position during treatment planning for dynamic‐shield IMBT of rectal cancer and to investigate a robust optimization method to account for these uncertainties. Important differences with the previous study include the change of treatment site, from prostate to rectum, and the change of applicator type, from multichannel interstitial to single channel intracavitary. Furthermore, more error scenarios were considered here as both rotational and translational uncertainties were included.

## 2 METHODS

### 2.1 Rectal IMBT shield

**FIGURE 1:**
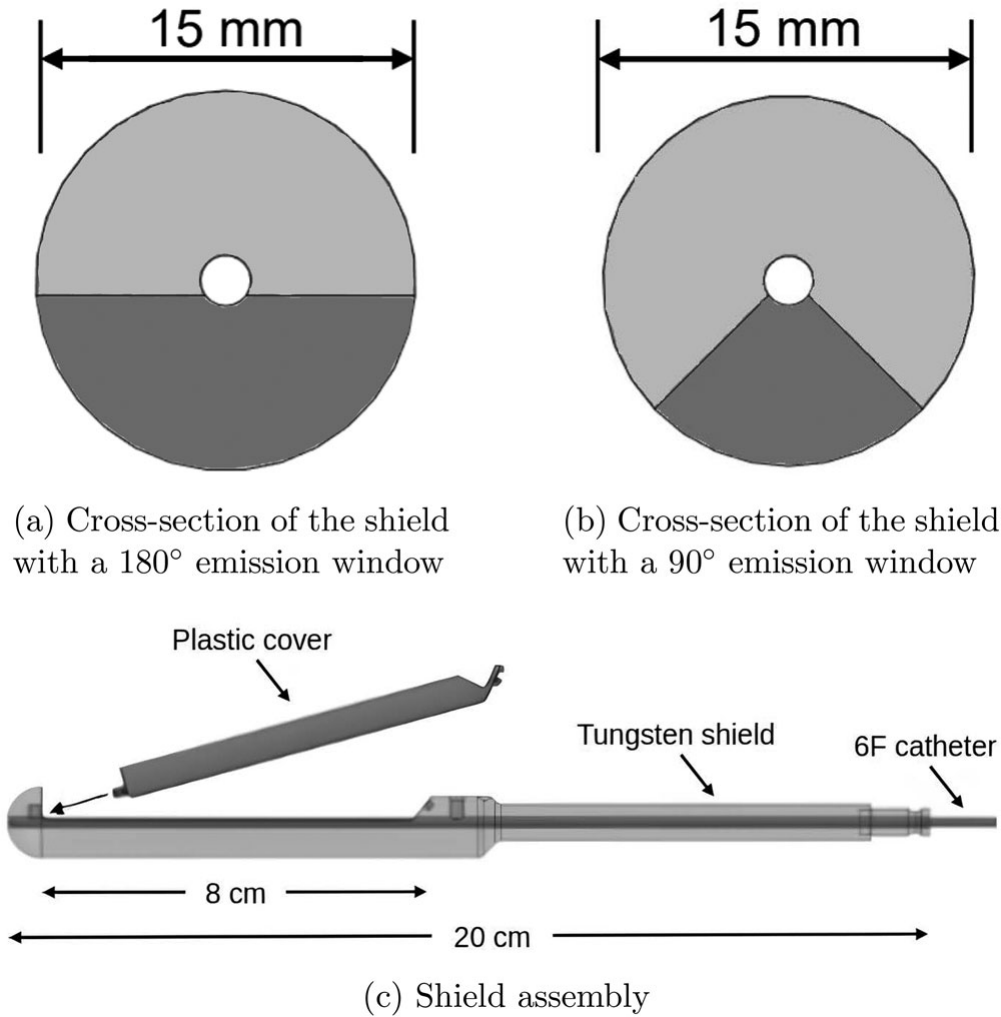
Cross‐sections of the shields with (a) $180^{\circ }$ and (b) $90^{\circ }$ emission windows. The assembly is shown in subfigure (c). The tungsten shield (light grey) features a groove on one end and a clip hole on the other, allowing the plastic part (dark grey) to snap into place.

A flexible silicone cylindrical applicator with an outer diameter of 20mm and an inner diameter of 15mm was used. A tungsten shield with an outer diameter of 15mm was inserted inside the applicator. A central 2mm source channel permitted the insertion a 6F catheter. Previously, it was found that $180^{\circ }$ and $90^{\circ }$ emission windows could be used for dynamic‐shield IMBT depending on the tumor size. Therefore, we included both shield designs in this study. In each case, the emission window had a length of 8cm as shown in Figure 1. The shield can be connected to a rotating delivery system.

During treatment planning, dwell positions were created along the source channel at 5mm intervals, and the shield rotation was limited to $15^{\circ }$ increments. A generic 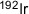 source was used.^32^

### 2.2 Monte carlo simulations

The same Geant4^33^, ^34^ based treatment planning system, RapidBrachyMCTPS,^17^, ^18^ as utilized in the previous study by Morén et al.^31^ was employed. The software includes a Monte Carlo‐based dose calculation engine and a graphical user interface allowing users to create treatment plans. The software is also equipped with a comprehensive dose analysis package. Different optimization models are implemented into the treatment planning system.^16^, ^35^ In this study, planning CT images were imported into RapidBrachyMCTPS and converted to voxelized phantoms in egsphant format. Patient tissue was approximated to water with unit mass density, while tungsten was assigned to the shield. Treatment planning was performed in two steps. The dose distribution for each dwell position and shield angle combination was first calculated with 3 × 3 × 3 mm^3^ voxels and 10^6^ histories. These were then used as input for the optimization algorithm. The initial penalty weights were the same for all patients but were fine‐tuned until an acceptable plan was achieved, that is, theclinical target volume (CTV) $D_{90}$ value was as close as possible to the prescription dose of 10 Gy. More specifically, the penalty weights for the target were increased, while those for the OAR were decreased if the CTV $D_{90}$ was less than 10 Gy, and vice versa. This method is similar to what is done in clinical practice. Automatic penalty weight tuning can be used in the future to standardize and accelerate this process.^36^ The resulting dose distribution was recalculated with 1 × 1 × 1 mm^3^ voxels and 10^8^ histories to achieve a statistical uncertainty of less than 1% at the 100% isodose line. Based on recommendations from the AAPM task group 268 (TG‐268),^37^ details about the Monte Carlo simulations can be found in the appendix.

### 2.3 Patient cohort

The ethics committee of the McGill University teaching hospitals, the Jewish General Hospital and the McGill University Health Center, approved this retrospective study. The patient data set consisted of ten rectal cancer patients' first HDR BT fraction. For planning purposes, we used contours of the CTV, the contralateral healthy rectal wall (contra), and two artificially defined box structures drawn 1cm above and below the superior/inferior extent of the tumor, referred to as the superior (sup) and the inferior (inf) dose spill regions.

### 2.4 Experimental design

Two scenarios were explored to investigate the effects of positional uncertainties: systematic errors of the shield rotation and systematic errors of the source translational position. They were compared to a scenario with no errors, referred to as the nominal scenario. Two main sources of error in the shield rotation were identified: the initial misalignment of the shield with the reference position ($0^{\circ }$) and the inherent uncertainties of the rotating system. Similarly, two sources of error in the source position were determined: the displacement of the shield between imaging and treatment, as well as the systemic effects of afterloader accuracy.

First, the dwell times were obtained by minimizing a quadratic penalty model (QPM), a common optimization model for treatment planning. This model served as a reference, and no uncertainties were considered. Secondly, a robust extension of QPM, referred to as QPM‐R, was implemented to take uncertainties into account. We evaluated two versions of QPM‐R that considered systematic errors of the shield rotation and source translational position separately. For shield rotation errors, two scenarios with systematic offsets of $\pm 5^{\circ }$ were included in QPM‐R, while for translational errors, two scenarios with offsets of ±2 mm were included. Scenarios with rotational errors up to $\pm 10^{\circ }$ and translational errors up to ±3 mm were examined to evaluate the obtained treatment plans. The Wilcoxon signed‐rank test was used to compare DVH metrics achieved with QPM and QPM‐R. A *p*‐value of 0.05 or less was considered significant.

### 2.5 Optimization model

Previously, a linear penalty model (LPM) and its robust version (LPM‐R) were proposed.^31^ Authors also tested QPM and its robust counterpart QPM‐R and presented the mathematical formulation in the Appendix. In this study, QPM and QPM‐R were used (Equations 1a‐1h). The notation is provided in Table 1.

**TABLE 1:** Indices, sets, parameters, and variables in the optimization model.

Indices	
i	Index for a dose point
j	Index for a combination of a dwell position and shield angle
s	Index for a structure
r	Index for a scenario
Sets	
S	Set of structures
$P_{s}$	Set of dose points in structure s∈S
J	Set of dwell position and shield angle combinations
R	Set of scenarios
Parameters	
$d_{ij}^{r}$	Dose‐rate contribution from dwell position and shield angle j∈J
	to dose point $i\in \cup_{s\in S}P_{s}$ in scenario r∈R
$L^{s}$	Prescription dose or lower dose bound for structure s∈S
$U^{s}$	Upper dose bound for structure s∈S
$w_{s}^{l}$	Non‐negative penalty for dose being too low in structure s∈S
$w_{s}^{u}$	Non‐negative penalty for dose being too high in structure s∈S
$q_{s}^{l}$	Non‐negative quadratic penalty for dose being too low in structure s∈S
$q_{s}^{u}$	Non‐negative quadratic penalty for dose being too high in structure s∈S
Variables	
$t_{j}$	Dwell time for dwell position and shield angle j∈J
$x_{i}^{rl}$	Penalty variable for dose being too low at dose point $i\in P_{s},\;s\in S$, in scenario r∈R
$x_{i}^{ru}$	Penalty variable for dose being too high at dose point $i\in P_{s},\;s\in S$, in scenario r∈R
$z^{r}$	Objective function value for scenario r∈R
$z^{w}$	Objective function value for the scenario with the worst‐case outcome

(1a) $$\begin{array}{c} \min z^{w}+\frac{1}{\left|R\right|}\sum_{r\in R}z^{r} \end{array}$$

(1b) $$\begin{array}{c} \text{subject to}\\ \sum_{s\in S}\sum_{i\in P_{s}}\left(w_{s}^{l}x_{i}^{\mathrm{rl}}+q_{s}^{l}{\left(x_{i}^{\mathrm{rl}}\right)}^{2}+w_{s}^{u}x_{i}^{\mathrm{ru}}+q_{s}^{u}{\left(x_{i}^{\mathrm{ru}}\right)}^{2}\right)\leq \;z^{r}\;r\in R \end{array}$$

(1c) $$\begin{array}{ccc} z^{r}\leq \; & z^{w} & r\in R \end{array}$$

(1d) $$\begin{array}{ccc} \sum_{j\in J}d_{ij}^{r}t_{j}\geq \; & L^{s}-x_{i}^{rl} & i\in P_{s},s\in S,r\in R \end{array}$$

(1e) $$\begin{array}{ccc} \sum_{j\in J}d_{ij}^{r}t_{j}\leq \; & U^{s}+x_{i}^{ru} & i\in P_{s},s\in S,r\in R \end{array}$$

(1f) $$\begin{array}{ccc} x_{i}^{rl}\geq & \;0 & i\in P_{s},s\in S,r\in R \end{array}$$

(1g) $$\begin{array}{ccc} x_{i}^{ru}\geq & \;0 & i\in P_{s},s\in S,r\in R \end{array}$$

(1h) $$\begin{array}{ccc} t_{j}\geq & \;0 & j\in J \end{array}$$

The objective function (1a) represents a weighted sum that minimizes the penalty associated with the worst‐case scenario and the average penalty across all scenarios. Constraints (1b) ensure proper assignment of penalty values for each scenario, while constraints (1c) guarantee that $z^{w}$ takes the penalty of the worst‐case scenario. Additionally, constraints (1d) and (1f) are used to model penalties for dose points with insufficient dose, while (1e) and (1g) handle penalties for dose points with excessive dose. Finally, constraints (1h) enforce the nonnegativity of dwell times.

The model QPM‐R belongs to the category of minimax stochastic programming.^22^ It was chosen as a compromise between minimax optimization, which only accounts for the worst‐case scenario, and stochastic programming, which only accounts for the average penalty. Considering several probability distributions, the expected objective function value was minimized across all scenarios. Both QPM and QPM‐R are convex models. The software Gurobi, version 9.5.1,^38^ was used for solving the optimization models.

In addition to the model QPM‐R, we have also investigated a variant referred to as the voxel‐wise worst‐case method.^22^ Instead of evaluating each scenario separately, a single value of the dose‐rate contributions was considered per dose point: the minimum dose‐rate contribution among all scenarios was used for dose points in the target, while the maximum dose‐rate contribution was used for dose points in the OARs.

## 3 RESULTS

Results are based on the evaluation of the final dose map calculated with 1 ${\mathrm{mm}}^{3}$ voxels and for dwell times rescaled to obtain a CTV $D_{90}$ value equal to the prescription dose of 10 Gy.

Table 2 shows the relative dosimetric indices obtained with QPM in the worst‐case scenario with $\pm 5^{\circ }$ rotational errors for the $180^{\circ }$ shield. For the CTV and the sup and inf dose spill regions, the differences from the nominal scenario were relatively small for all patients (≤ 4%). Comparatively, the contra $D_{0.1cc}$ increased by up to 15%. However, the $D_{2cc}$ and $D_{50}$ of this structure increased by only 3% and 1%, respectively, on average. Similarly, Table 3 presents the relative dosimetric indices obtained with QPM in the worst‐case scenario with $\pm 5^{\circ }$ rotational errors, but for the $90^{\circ }$ shield. Again, there was no significant change in the CTV $D_{90}$ compared to the nominal scenario. However, the CTV $D_{98}$ decreased by at least 10% in three patients. The sup and inf dose spill regions were relatively unaffected by the rotational error, but the contralateral rectal wall $D_{0.1cc}$ increased by 14% on average with a maximal change of 32%. An increase of 7% of the $D_{2cc}$ was observed for one patient, but the mean $D_{50}$ only increased by 1%.

**TABLE 2:** Dosimetric index ratios (worst‐case versus nominal scenario) obtained with the $180^{\circ }$ shield for each patient with model QPM for $\pm 5^{\circ }$ rotational errors.

	CTV			sup			inf			contra		
Patient	Vol (cc)	$D_{90}$	$D_{98}$	$D_{0.1cc}$	$D_{2cc}$	$D_{50}$	$D_{0.1cc}$	$D_{2cc}$	$D_{50}$	$D_{0.1cc}$	$D_{2cc}$	$D_{50}$
P1	14.5	0.99	0.96	1.02	1.01	1.01	1.00	1.01	1.01	1.13	1.02	1.00
P2	11.0	1.00	0.99	1.00	1.00	1.00	1.00	1.00	1.00	1.06	1.01	1.00
P3	13.9	0.99	0.97	1.00	1.00	1.04	1.00	1.00	1.02	1.15	1.05	1.01
P4	5.1	0.99	0.98	1.02	1.02	1.01	1.01	1.01	1.02	1.03	1.03	1.01
P5	11.3	0.99	0.98	1.00	1.00	1.00	1.00	1.01	1.00	1.08	1.01	1.00
P6	6.1	0.99	0.99	1.00	1.00	1.00	1.00	1.00	1.01	1.10	1.02	1.01
P7	11.6	0.99	0.99	1.00	1.00	1.04	1.00	1.00	1.04	1.13	1.04	1.01
P8	11.4	0.99	0.99	1.00	1.00	1.01	1.01	1.00	1.00	1.10	1.03	1.02
P9	9.5	1.00	1.00	1.00	1.01	1.02	1.01	1.01	1.00	1.12	1.05	1.00
P10	10.5	0.99	0.99	1.00	1.01	1.01	1.00	1.00	1.00	1.14	1.04	1.00
Mean	10.5	0.99	0.98	1.00	1.00	1.01	1.00	1.00	1.01	1.11	1.03	1.01

Abbreviations: CTV, clinical target volume; inf, inferior; QPM, quadratic penalty model; sup, superior.

**TABLE 3:** Dosimetric index ratios (worst‐case versus nominal scenario) obtained with the $90^{\circ }$ shield for each patient with model QPM for $\pm 5^{\circ }$ rotational errors.

	CTV			sup			inf			contra		
Patient	Vol (cc)	$D_{90}$	$D_{98}$	$D_{0.1cc}$	$D_{2cc}$	$D_{50}$	$D_{0.1cc}$	$D_{2cc}$	$D_{50}$	$D_{0.1cc}$	$D_{2cc}$	$D_{50}$
P1	14.5	0.99	0.97	1.00	1.00	1.00	1.00	1.00	1.00	1.21	1.03	1.00
P2	11.0	0.98	0.89	1.00	1.00	1.00	1.00	1.00	1.00	1.10	1.03	1.01
P3	13.9	0.99	0.96	1.01	1.00	1.02	1.01	1.00	1.01	1.18	1.04	1.01
P4	5.1	0.99	0.96	1.03	1.02	1.00	1.01	1.02	1.04	1.06	1.07	1.01
P5	11.3	0.95	0.84	1.00	1.00	1.01	1.00	1.01	1.00	1.09	1.02	1.01
P6	6.1	0.99	0.98	1.00	1.00	1.00	1.00	1.00	1.01	1.08	1.01	1.00
P7	11.6	0.99	0.98	1.01	1.00	1.03	1.01	1.01	1.01	1.12	1.03	1.00
P8	11.4	0.98	0.90	1.01	1.00	1.01	1.00	1.00	1.00	1.20	1.01	1.01
P9	9.5	0.98	0.96	1.01	1.01	1.01	1.00	1.01	1.00	1.32	1.02	1.00
P10	10.5	0.98	0.98	1.01	1.00	1.01	1.02	1.01	1.01	1.11	1.04	1.01
Mean	10.5	0.98	0.95	1.01	1.00	1.01	1.00	1.00	1.01	1.14	1.03	1.01

Abbreviations: CTV, clinical target volume; inf, inferior; QPM, quadratic penalty model; sup, superior.

Tables 4 and 5 list the relative dosimetric indices with the $180^{\circ }$ and $90^{\circ }$ shields, respectively, for all patients in the worst‐case scenario with ±2 mm translational errors of the source for treatment plans obtained with QPM. The differences from the nominal scenario were greater in all structures except the contralateral rectal wall when compared to rotational errors. For each patient, there was a large increase in absorbed dose to at least one of the dose spill regions, but how the dose was increased and which of the structures was most affected depended on the patient‐specific anatomy. This was expected as the translational source position errors would decrease the distance to either of the dose spill regions. The results were similar for both shield designs.

**TABLE 4:** Dosimetric index ratios (worst‐case versus nominal scenario) obtained with the $180^{\circ }$ shield for each patient with model QPM for ±2mm translational errors.

	CTV			sup			inf			contra		
Patient	Vol (cc)	$D_{90}$	$D_{98}$	$D_{0.1cc}$	$D_{2cc}$	$D_{50}$	$D_{0.1cc}$	$D_{2cc}$	$D_{50}$	$D_{0.1cc}$	$D_{2cc}$	$D_{50}$
P1	14.5	0.96	0.95	1.12	1.08	1.12	1.20	1.14	1.11	1.14	1.02	1.00
P2	11.0	0.97	0.95	1.21	1.17	1.07	1.24	1.15	1.08	1.03	1.02	1.00
P3	13.9	0.98	0.94	1.20	1.14	1.10	1.18	1.14	1.10	1.17	1.04	1.00
P4	5.1	0.98	0.97	0.98	1.03	1.02	1.21	1.14	1.12	1.02	1.01	1.00
P5	11.3	0.85	0.85	1.16	1.15	1.15	1.87	1.21	1.09	1.10	1.03	1.03
P6	6.1	0.94	0.92	0.97	1.01	1.17	1.25	1.15	1.11	1.02	0.99	1.01
P7	11.6	0.97	0.94	1.18	1.13	1.11	1.18	1.13	1.12	1.01	1.00	1.00
P8	11.4	0.96	0.95	1.07	1.07	1.09	1.22	1.17	1.13	1.02	1.00	1.01
P9	9.5	0.97	0.95	1.21	1.14	1.08	1.21	1.14	1.11	1.04	1.00	1.01
P10	10.5	0.93	0.89	1.16	1.13	1.11	1.17	1.13	1.12	1.04	1.01	1.00
Mean	10.5	0.95	0.93	1.13	1.11	1.10	1.26	1.15	1.11	1.06	1.01	1.00

Abbreviations: CTV, clinical target volume; inf, inferior; QPM, quadratic penalty model; sup, superior.

**TABLE 5:** Dosimetric index ratios (worst‐case versus nominal scenario) obtained with the $90^{\circ }$ shield for each patient with model QPM for ±2 mm translational errors.

	CTV			sup			inf			contra		
Patient	Vol (cc)	$D_{90}$	$D_{98}$	$D_{0.1cc}$	$D_{2cc}$	$D_{50}$	$D_{0.1cc}$	$D_{2cc}$	$D_{50}$	$D_{0.1cc}$	$D_{2cc}$	$D_{50}$
P1	14.5	0.99	0.96	1.21	1.14	1.14	1.18	1.13	1.10	1.01	1.00	1.00
P2	11.0	0.98	0.96	1.19	1.15	1.08	1.23	1.14	1.08	1.03	1.01	0.99
P3	13.9	0.98	0.96	1.22	1.14	1.09	1.19	1.14	1.07	1.16	1.04	1.00
P4	5.1	0.98	0.96	1.11	1.03	1.18	1.24	1.15	1.14	1.03	1.05	1.01
P5	11.3	0.86	0.87	1.19	1.15	1.14	1.86	1.16	1.14	1.01	1.02	1.04
P6	6.1	0.95	0.92	1.04	1.07	1.15	1.23	1.16	1.16	1.03	1.01	1.00
P7	11.6	0.97	0.96	1.18	1.13	1.14	1.18	1.14	1.13	1.04	1.01	1.00
P8	11.4	0.96	0.95	1.09	1.08	1.14	1.22	1.16	1.13	1.02	1.00	1.01
P9	9.5	0.97	0.94	1.20	1.14	1.11	1.22	1.15	1.14	1.02	1.01	1.01
P10	10.5	0.95	0.90	1.17	1.13	1.11	1.18	1.13	1.10	0.99	1.01	1.00
Mean	10.5	0.96	0.94	1.17	1.12	1.12	1.27	1.14	1.12	1.04	1.02	1.01

Abbreviations: CTV, clinical target volume; inf, inferior; QPM, quadratic penalty model; sup, superior.

Models QPM and QPM‐R are compared in Tables 6, 7, 8, and 9. There were only small differences between the two models for the OAR dosimetric indices. For the $180^{\circ }$ shield, a statistically significant difference between the optimization models was observed for the contra $D_{0.1cc}$ for all rotational error values. The inf $D_{2cc}$ also significantly differed for $10^{\circ }$ errors. For the $90^{\circ }$ shield, only the sup $D_{2cc}$ was found to be significantly different for $3^{\circ }$ rotational errors. Although not statistically significant, a 6% and 8% improvement in inf $D_{0.1cc}$ was observed with QPM‐R for translational errors of ±2 mm and ±3 mm, respectively, with the $180^{\circ }$ shield. Similarly, a 4% and 7% improvement of this DVH metric was observed with the $90^{\circ }$ shield. Additionally, these tables demonstrate that the magnitude of changes in DVH metrics grew as the rotational or translational errors increased.

**TABLE 6:** Mean dosimetric index ratios (worst‐case versus nominal scenario) obtained with the $180^{\circ }$ shield for $\pm 3^{\circ }$, $\pm 5^{\circ }$, and $\pm 10^{\circ }$ rotational errors for models QPM and QPM‐R. An indicates that there is a significant difference between QPM and QPM‐R.

		CTV		sup			inf			contra		
Model	Error	$D_{90}$	$D_{98}$	$D_{0.1cc}$	$D_{2cc}$	$D_{50}$	$D_{0.1cc}$	$D_{2cc}$	$D_{50}$	$D_{0.1cc}$	$D_{2cc}$	$D_{50}$
QPM	$3^{\circ }$	1.00	0.99	1.00	1.00	1.01	1.00	1.00	1.00	1.05	1.01	1.00
QPM‐R	$3^{\circ }$	1.00	0.99	1.00	1.00	1.01	1.00	1.00	1.00	1.07 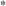	1.01	1.00
QPM	$5^{\circ }$	0.99	0.98	1.00	1.00	1.01	1.00	1.00	1.01	1.11	1.03	1.01
QPM‐R	$5^{\circ }$	0.99	0.98	1.01	1.00	1.01	1.00	1.00	1.00	1.12 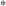	1.03	1.01
QPM	$10^{\circ }$	0.98	0.93	1.01	1.01	1.02	1.01	1.01	1.01	1.26	1.09	1.01
QPM‐R	$10^{\circ }$	0.98	0.94	1.01	1.01	1.02	1.00	1.01 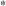	1.01	1.28 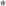	1.08	1.02

Abbreviations: CTV, clinical target volume; inf, inferior; QPM, quadratic penalty model; QPM‐R, quadratic penalty model‐robust; sup, superior.

**TABLE 7:** Mean dosimetric index ratios (worst‐case versus nominal scenario) obtained with the $90^{\circ }$ shield for $\pm 3^{\circ }$, $\pm 5^{\circ }$, and $\pm 10^{\circ }$ rotational errors for models QPM and QPM‐R. An indicates that there is a significant difference between QPM and QPM‐R.

		CTV		sup			inf			contra		
Model	Error	$D_{90}$	$D_{98}$	$D_{0.1cc}$	$D_{2cc}$	$D_{50}$	$D_{0.1cc}$	$D_{2cc}$	$D_{50}$	$D_{0.1cc}$	$D_{2cc}$	$D_{50}$
QPM	$3^{\circ }$	0.99	0.97	1.00	1.00	1.01	1.00	1.00	1.00	1.08	1.02	1.00
QPM‐R	$3^{\circ }$	0.99	0.98	1.00	1.00 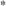	1.01	1.00	1.00	1.00	1.08	1.02	1.01
QPM	$5^{\circ }$	0.98	0.95	1.01	1.00	1.01	1.00	1.00	1.01	1.14	1.03	1.01
QPM‐R	$5^{\circ }$	0.98	0.95	1.01	1.00	1.01	1.00	1.01	1.00	1.14	1.03	1.01
QPM	$10^{\circ }$	0.95	0.84	1.01	1.01	1.02	1.01	1.01	1.01	1.32	1.09	1.02
QPM‐R	$10^{\circ }$	0.95	0.86	1.01	1.01	1.02	1.01	1.01	1.01	1.31	1.09	1.02

Abbreviations: CTV, clinical target volume; inf, inferior; QPM, quadratic penalty model; QPM‐R, quadratic penalty model‐robust; sup, superior.

**TABLE 8:** Mean dosimetric index ratios (worst‐case versus nominal scenario) obtained with the $180^{\circ }$ shield for ±1, ±2, and ±3mm translational errors for models QPM and QPM‐R. An indicates that there is a significant difference between QPM and QPM‐R.

		CTV		sup			inf			contra		
Model	Error	$D_{90}$	$D_{98}$	$D_{0.1cc}$	$D_{2cc}$	$D_{50}$	$D_{0.1cc}$	$D_{2cc}$	$D_{50}$	$D_{0.1cc}$	$D_{2cc}$	$D_{50}$
QPM	1mm	0.98	0.97	1.06	1.05	1.05	1.12	1.07	1.05	1.03	1.01	1.00
QPM‐R	1mm	0.98	0.97	1.06	1.05	1.05	1.09	1.07	1.05	1.03	1.01	1.00
QPM	2mm	0.95	0.93	1.13	1.11	1.10	1.26	1.15	1.11	1.06	1.01	1.00
QPM‐R	2mm	0.96	0.93	1.12	1.10	1.10	1.20	1.14	1.10	1.05	1.01	1.00
QPM	3mm	0.92	0.88	1.21	1.16	1.15	1.44	1.23	1.16	1.08	1.02	1.00
QPM‐R	3mm	0.92	0.88	1.21	1.16	1.15	1.36	1.21	1.16	1.07	1.02	1.00

Abbreviations: CTV, clinical target volume; inf, inferior; QPM, quadratic penalty model; QPM‐R, quadratic penalty model‐robust; sup, superior.

**TABLE 9:** Mean dosimetric index ratios (worst‐case versus nominal scenario) obtained with the $90^{\circ }$ shield for ±1, ±2, and ±3mm translational errors for models QPM and QPM‐R. An indicates that there is a significant difference between QPM and QPM‐R.

		CTV		sup			inf			contra		
Model	Error	$D_{90}$	$D_{98}$	$D_{0.1cc}$	$D_{2cc}$	$D_{50}$	$D_{0.1cc}$	$D_{2cc}$	$D_{50}$	$D_{0.1cc}$	$D_{2cc}$	$D_{50}$
QPM	1mm	0.98	0.98	1.07	1.06	1.06	1.12	1.07	1.05	1.02	1.01	1.00
QPM‐R	1mm	0.99	0.98	1.08	1.06	1.06	1.10	1.06	1.06	1.02	1.01	1.00
QPM	2mm	0.96	0.94	1.17	1.12	1.12	1.27	1.14	1.12	1.04	1.02	1.01
QPM‐R	2mm	0.97	0.95	1.17	1.12	1.13	1.23	1.14	1.12	1.05	1.02	1.00
QPM	3mm	0.93	0.89	1.27	1.19	1.19	1.47	1.23	1.19	1.06	1.02	1.00
QPM‐R	3mm	0.94	0.90	1.28	1.19	1.19	1.40	1.22	1.18	1.08	1.02	1.00

Abbreviations: CTV, clinical target volume; inf, inferior; QPM, quadratic penalty model; QPM‐R, quadratic penalty model‐robust; sup, superior.

**FIGURE 2:**
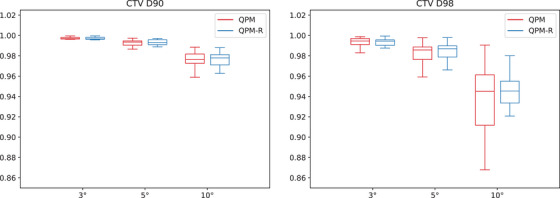
Mean dosimetric index ratios (worst‐case versus nominal scenario) for the CTV obtained with the $180^{\circ }$ shield $\pm 3^{\circ }$, $\pm 5^{\circ }$, and $\pm 10^{\circ }$ rotational errors for models QPM (red) and QPM‐R (blue). CTV, clinical target volume; QPM, quadratic penalty model; QPM‐R, quadratic penalty model‐robust.

**FIGURE 3:**
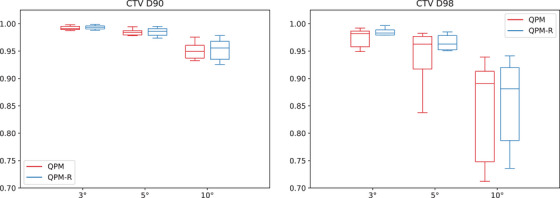
Mean dosimetric index ratios (worst‐case versus nominal scenario) for the CTV obtained with the $90^{\circ }$ shield $\pm 3^{\circ }$, $\pm 5^{\circ }$, and $\pm 10^{\circ }$ rotational errors for models QPM (red) and QPM‐R (blue). CTV, clinical target volume; QPM, quadratic penalty model; QPM‐R, quadratic penalty model‐robust.

**FIGURE 4:**
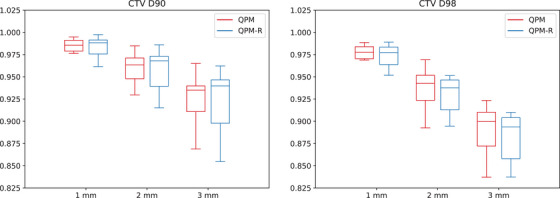
Mean dosimetric index ratios (worst‐case versus nominal scenario) for the CTV obtained with the $180^{\circ }$ shield ±1, ±2, and ±3 mm translational errors for models QPM (red) and QPM‐R (blue). CTV, clinical target volume; QPM, quadratic penalty model; QPM‐R, quadratic penalty model‐robust.

**FIGURE 5:**
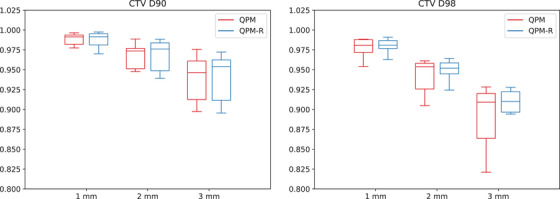
Mean dosimetric index ratios (worst‐case versus nominal scenario) for the CTV obtained with the $90^{\circ }$ shield ±1, ±2, and ±3 mm translational errors for models QPM (red) and QPM‐R (blue). CTV, clinical target volume; QPM, quadratic penalty model; QPM‐R, quadratic penalty model‐robust.

Figures 2, 3, 4 and 5 present the distribution of the CTV $D_{90}$ and $D_{98}$ across all patients for QPM and QPM‐R. The spread of values generally increased with the magnitude of the error, suggesting that the effects of positional errors on dosimetry were more influenced by patient anatomy for larger errors.

There was an increase in the number of active dwell positions (dwell times greater than 0.5 s) with the robust model. For the $180^{\circ }$ shield, the mean number of active dwell positions per plan increased from 7.4 with QPM to 8.3 and 7.6 with QPM‐R with rotational and translational errors, respectively. For the $90^{\circ }$ shield, this value increased from 13.0 with QPM to 16.1 with both versions of QPM‐R. Moreover, the longest dwell time for the $180^{\circ }$ shield was, on average, reduced by 11% and 5% with QPM‐R accounting for rotational and translational errors. For the $90^{\circ }$ shield, the longest dwell time was reduced by 7% with QPM‐R with rotational errors and increased by 1% with QPM‐R with translational errors. The difference in total treatment times between models QPM and QPM‐R was less than 0.2% for both rotational and translational errors.

Preliminary results with the voxel‐wise worst‐case method were considerably worse than for QPM and QPM‐R, for both the nominal scenario and the scenarios with errors, and are not included here. It appeared that the voxel‐wise worst dose‐rate contribution gave a too‐pessimistic estimate to be used for treatment planning with the studied shield designs.

## 4 DISCUSSION

In this study, we assessed the impact of rotational shield angle and translational source position errors on the dose distribution when using the commonly employed QPM model in the context of treatment planning for rectal IMB. Furthermore, planning was performed with QPM‐R to investigate to what extent the uncertainties can be mitigated with a robust optimization model if scenarios with rotational shield errors or translational source errors are included in the treatment planning. Our approach is flexible since the user can decide a priority between the worst scenario and the nominal scenario.

In the scenario with $\pm 5^{\circ }$ rotational errors, the mean CTV $D_{90}$ decreased by 1%‐2% compared to the nominal scenario for all but one patient, as presented in Tables 2 and 3. Rotational errors had a greater impact on the contralateral rectal wall, with an average increase of 11% and 14% to the $D_{0.1cc}$ with the $180^{\circ }$ and $90^{\circ }$ shields. However, the difference in absorbed dose to larger volumes of the contralateral rectum was smaller (3% and 1% for the $D_{2cc}$ and $D_{50}$, respectively). This suggests that rotational errors result in more intense hot spots in the contralateral rectal wall, but the average dose to the structure is less affected. Furthermore, the shield with a smaller emission window was more sensitive to rotational errors because these errors caused a larger proportional deviation in dose coverage, especially for the CTV $D_{98}$.

In the case of ±2mm translational source position errors, an increase in absorbed dose to all OARs was observed, as illustrated in Tables 4 and 5. Moreover, there were larger variations in dosimetric indices among patients, except for the contralateral healthy rectal wall. Notably, for the $180^{\circ }$ shield with ±2mm errors, the relative CTV $D_{90}$ ranged from 85% to 98%, whereas it fluctuated between 99% and 100% for $\pm 5^{\circ }$ rotational errors. Additionally, the relative $D_{0.1cc}$ values varied by 23% and 70% between patients for the sup and inf dose spill regions for translational errors of ±2mm, compared to just 2% and 1% for $\pm 5^{\circ }$ rotational errors. Similar trends were observed with the $90^{\circ }$ shield. The inf dose spill region $D_{0.1cc}$ increased by over 85% for Patient 5 due to ±2mm translational errors (Tables 4 and 5). This was because the OAR was drawn closer to the tumor, which was located near the inferior border of the CT scan. As a result, the OAR was positioned in a steeper dose gradient, making it more susceptible to translational errors compared to other patients. This highlights that the magnitude of dose changes depends on the anatomy of the patient.

As local excision and nonoperative treatment replace total mesorectal excision for patients with complete response, it is important to accurately assess the surrounding healthy rectal dose. Our results, shown in Tables 6, 7, 8 and 9, indicate a steep increase in the dose to OAR as positional errors grow. In particular, the contra $D_{0.1cc}$ roughly doubled when the shield rotational error increased from $\pm 3^{\circ }$ to $\pm 5^{\circ }$. The effect was even more pronounced for translational errors as nearly all OAR metrics doubled when displacement increased from ±1 to ±2mm. However, additional work is needed to determine the clinical significance of the differences in dose metrics caused by positional uncertainties. Nevertheless, this study provides valuable information for quality assurance protocols. Based on these findings, we recommend keeping rotational errors below $\pm 3^{\circ }$ and translational errors below ±1mm.

In treatment plans obtained with QPM‐R, there were more active dwell positions than in plans obtained with QPM, and the longest dwell times were shorter. These are properties that are considered advantageous. Dwell time modulation restrictions are commonly applied in treatment planning for prostate cancer to obtain more homogeneous dwell times.^39^ This avoids high‐dose volumes around dwell positions with long dwell times. The proposed robust optimization approach is a flexible way to achieve both homogeneous dwell times and a robust treatment plan against uncertainties for rectal IMBT. Furthermore, it could also be useful for conventional HDR BT. Minimum deliverable dwell times are not incorporated into QPM‐R. In practice, the treatment planning system will round the dwell times based on the precision of the afterloader. We estimate this will have a negligible effect on the final dose distribution.

Improvements with model QPM‐R were small in terms of dosimetric indices, and our results indicate that the potential benefit of incorporating different scenarios into treatment planning is limited. A statistically significant increase of the contra $D_{0.1cc}$ was observed with QPM‐R for rotational errors with the $180^{\circ }$. However, these increases remained within 2%. Moreover, it is difficult to draw definite conclusions from these statistical tests due to our small sample size. Our previous study evaluating the dosimetric effect of a robust optimization approach for prostate IMBT found that errors could be mitigated to a larger extent than for rectal cancer.^31^ One reason for this could be that there are more degrees of freedom in treatment planning for prostate cancer as there are positions in several catheters that can be combined. In the IMBT rectal applicator presented in this study, only one catheter is used; thus, there are fewer possibilities to compensate for uncertainties. However, QPM‐R incorporating scenarios with positional errors might be more advantageous for conventional rectal HDR BT, which uses multiple catheters. A new protocol for rectal IMBT treatment planning, including the addition of contoured dose spill regions for optimization, is under investigation. Before adding them to QPM‐R, appropriate constraints must be determined for these new regions of interest.

The GEC‐ESTRO BRAPHYQS (BRAchytherapy PHYsics Quality Assurances System) published a review of the different clinical brachytherapy uncertainties.^40^ Their report provided data obtained from afterloader manufacturers and presented source positioning uncertainties of 1mm. Several sources of uncertainty and their effects were evaluated in a prostate HDR BT study.^41^ The uncertainties were related to the dwell positions, dwell times or contoured structures (prostate, urethra and rectum). A worst‐case value of 2.47 mm was considered for the movement of dwell positions. Translational and rotational errors of the applicator reconstruction of ± 3mm and $\pm 15^{\circ }$, respectively, have been investigated for cervical cancer.^42^ The authors demonstrated a mean absorbed dose increase to the rectum and bladder $D_{0.1cc}$ of up to 6% per mm displacement. In comparison, our results using the QPM model showed increases per mm of up to 9%, 16% and 3% for the $D_{0.1cc}$ of the sup, inf and contralateral regions, respectively, as shown in Tables 8 and 9.

Webster et al.^11^ performed an uncertainty analysis for dynamically modulated brachytherapy for rectal cancer. For 3mm translational errors, the authors demonstrated that the CTV $D_{98}$ decreased by 12%, while our results for QPM‐R showed a similar drop of 12% and 10% with the $180^{\circ }$ and $90^{\circ }$ shield, respectively. For $5^{\circ }$ rotational errors, they observed an 8% reduction of the CTV $D_{98}$, while in this study for QPM‐R, a 2% and 5% decrease for $180^{\circ }$ and $90^{\circ }$ shield, respectively, was observed. This difference could be attributed to the smaller emission window used by Webster et al.,^11^ which makes it more sensitive to rotational errors. A study by Hopfensperger et al. presented the population percentile allowance method to evaluate the tolerance of IMBT to spatial uncertainties.^43^ They proposed this mathematical model as an alternative to robust optimization for determining the design constraints when developing IMBT applicators. The authors applied the method to the use case of helical rotating shield brachytherapy. They found that the tolerance for shield orientation and applicator position uncertainties were $\pm 4.25^{\circ }$ and ±1.0 mm, respectively, to ensure that 90% of patients had a maximum DVH uncertainty below their tolerance of 10%. The population percentile allowance method could be combined with an approach considering uncertainty in treatment planning, such as QPM‐R. The result would indicate a higher uncertainty tolerance if the uncertainties are mitigated.

In this work, an optimization model based on linear and quadratic penalties was used. An alternative is to explicitly include biological indices such as tumor control probability and normal tissue complication probability in the model. However, before such an approach can be beneficial, it is necessary to find accurate and validated approximations of the correlation between dose distribution and treatment outcomes. A model incorporating dosimetric indices, or approximations thereof, is another option, but established guidelines are essential for such models to be fully beneficial. An advantage of convex models such as the QPM and the QPM‐R is that they are generally faster to solve, and the convexity provides a level of reproducibility as a globally optimal solution can be obtained.

A limitation of this study was that rotational errors of the shield angle and translational errors of the source position were considered separately. However, both types of uncertainties exist simultaneously in the clinic. The proposed model QPM‐R could be extended to include both types of error scenarios in a future study. The approach based on quadratic penalties was chosen as it is fast, well‐studied and available in clinical treatment planning systems. Chance‐constrained programming can be used to ensure that, for example, constraints on dosimetric indices are satisfied with a certain probability, but such approaches were not considered in this work as they were deemed too computationally expensive.

## 5 CONCLUSIONS

We investigated the impact of rotational shield angle and translational source position errors on dose plan quality in rectal cancer IMBT. Rotational errors minimally affected the dose to the CTV, but errors greater than $3^{\circ }$ increased the dose to the contralateral $D_{0.1cc}$. The $90^{\circ }$ emission window shield was more sensitive to rotational errors than the $180^{\circ }$ shield. Translational errors resulted in cold spots within the CTV for certain patients. They also lead to an important increase in the dose to the sup and inf dose spill regions for both shield designs. The robust optimization approach had a limited impact on mitigating positional errors. This underscores the importance of stringent quality assurance protocols, particularly for translational source position errors, which should ideally be kept within 1mm.

## CONFLICT OF INTEREST STATEMENT

The authors declare no conflicts of interest. 

## Monte carlo calculations

See Table A1 for details about the Monte Carlo simulations, according to recommendations of TG‐268.^37^

**TABLE A1:** Monte Carlo simulation methods used to calculate the dose‐rate contributions for this work. The simulation methods are based on the recommendations of TG‐268.

Item name	Description
Code, version	RapidBrachyMCTPS,^17^, ^18^ Geant4 10.2^33^, ^34^
Validation	Previously validated^17^
timing	Up to 5 min per dwell position on a 2 × AMD Rome 7532 2.40 GHz 256M cache L3.
Geometry	Voxelized geometry (egsphant) extracted from DICOM CT images and DICOM RT Structure Set files. Voxel size: for dwell time optimization a) 3 x 3 x 3 ${\mathrm{mm}}^{3}$ and for final dose calculation b) 1 x 1 x 1 ${\mathrm{mm}}^{3}$.
Materials	Patient geometry estimated to be water equivalent with unit mass density, with the density of the shields taken into account.
Source description	MicroSelectron v2 source geometry. Explicit simulation of radioactive decay spectra from ENSDF.^44^, ^45^ Source positions and orientation imported from DICOM RT Plan files.
Cross sections	EPDL97, EEDL97, EADL97.^46^, ^47^, ^48^
Transport parameters	PENELOPE low‐energy electromagnetic physics list with default transport parameters. Electron transport off. Production cut: 0.1 mm.^17^
Variance reduction technique	Tracklength estimator using mass‐energy absorption coefficient library provided in RapidBrachyMCTPS.^49^, ^50^
Scored quantities	Absorbed dose (collisional kerma approximation) scored to water. Voxel size: a) 3 x 3 x 3 ${\mathrm{mm}}^{3}$ and b) 1 x 1 x 1 ${\mathrm{mm}}^{3}$.
#histories & statistical uncertainties	$10^{8}$ decay events. Type A uncertainty for the final dose calculation is 0.6% on the 100% isodose line.
Statistical methods	History‐by‐history method.
Postprocessing	Dose to voxels converted into dose‐volume parameters using RapidBrachyMCTPS.
